# The protective effect of dronedarone on the structure and mechanical properties of the aorta in hypertensive rats by decreasing the concentration of symmetric dimethylarginine (SDMA)

**DOI:** 10.1371/journal.pone.0216820

**Published:** 2019-05-21

**Authors:** Begoña Quintana-Villamandos, María del Carmen González, María Jesús Delgado-Martos, Perla Yareli Gutiérrez-Arzapalo, Rainer H. Böger, Nicole Lüneburg, David Muñoz, Emilio Delgado-Baeza

**Affiliations:** 1 Department of Anesthesiology, Hospital Gregorio Marañón, Madrid, Spain; 2 Departament of Pharmacology and Toxicology, Faculty of Medicine, University Complutense de Madrid, Madrid, Spain; 3 Department of Physiology, Faculty of Medicine, University Autónoma of Madrid, Spain; 4 Molecular Biology Laboratory, Department Experimental Medicine and Surgery, Hospital Gregorio Marañón, Madrid, Spain; 5 Center of Research and Teaching in Health Sciences (CIDOCS), Autonomous University of Sinaloa, Sinaloa, Mexico; 6 Department of Clinical Pharmacology and Toxicology, University Medical Center Hamburg-Eppendorf, Hamburg, Germany; 7 Department of Experimental Surgery, University Autónoma of Madrid, Madrid, Spain; The University of Manchester, UNITED KINGDOM

## Abstract

**Background and aims:**

Dronedarone is a new multichannel-blocking antiarrhythmic for the treatment of patients with atrial fibrillation. Our group has demonstrated that dronedarone produces regression of cardiac remodeling; however, its effect on the remodeling of the elastic arteries has not yet been reported. We aim to assess the effects of dronedarone on the regression of thoracic aortic remodeling in spontaneously hypertensive rats (SHRs).

**Method:**

Ten-month-old male SHRs were randomly assigned to an intervention group (SHR-D), where the animals received dronedarone treatment (100 mg/kg), to a control group (SHR) where rats were given vehicle, or to a group (SHR-A) where they were given amiodarone. A fourth group of normotensive control rats (Wistar-Kyoto rats, WKY) was also added. After two weeks of treatment, we studied the structure, the elastic fiber content of the thoracic aorta using histological techniques and confocal microscopy, and the vascular mechanical properties using an organ bath and isometric tension analysis. A mass spectrometric determination of symmetric dimethylarginine (SDMA) concentrations was performed.

**Results:**

SHR group developed the classic remodeling expected from the experimental model: outward hypertrophic remodeling, increased elastic fiber content and wall stiffness. However, the SHR-D group showed statistically significantly lower values for aortic tunica media thickness, wall to lumen ratio, external diameter, cross-sectional area, volume density of the elastic fibers, wall stiffness, and aortic SDMA concentration when compared to the SHR group. These parameters were similar in the SHR and SHR-A groups. Interestingly, the values for tunica media thickness, volume density of the elastic fibers, wall stiffness, and SDMA concentration obtained from the SHR-D group were similar to those measured in the WKY group.

**Conclusion:**

These results suggest that dronedarone improves the structure and passive mechanical properties of the thoracic aorta in hypertensive rats, and that this protective effect could be associated with a reduction in the concentration of aortic SDMA.

## Introduction

Hypertension is the most common cause of hypertensive heart disease [1]. Hypertension induces cardiac remodeling, such as hypertrophy, which is related to arrhythmias (atrial and ventricular fibrillation), myocardial ischemia, and sudden cardiac death [2]. Hypertension increases the peripheral vascular resistance because of structural and functional changes to the large (conductive) and small (resistance) arteries [3]. However, the use of antihypertensive therapy can prevent cardiovascular events by producing a regression of cardiovascular remodeling [4–7].

Dronedarone is a new multichannel-blocking antiarrhythmic for the treatment of patients with atrial fibrillation [8]. The ATHENA trial has shown a significant reduction in the time to first cardiovascular hospitalization or death in patients with atrial arrhythmias receiving dronedarone [9]. Dronedarone produces a reduction in the risk of stroke [10] and acute coronary syndrome [11] that may be related to reduced heart rate and arterial blood pressure [12,13]. Our group has previously demonstrated that dronedarone produces regression of left ventricular hypertrophy (LVH) in hypertensive rats after two weeks of treatment (resulting in reduced left ventricular mass, changes in the cardiomyocytes and collagen of the left ventricle, and an improvement in cardiac metabolism) [14]. However, the impact of this drug on vascular remodeling has not yet been studied.

Symmetric dimethylarginine (SDMA) is a biomarker that indirectly reduces the synthesis of nitric oxide (NO) by inhibiting the cellular uptake of the NO precursor, L-arginine [15]. Some recent studies have suggested that SDMA is associated with cardiovascular events [16,17], and it has been identified as an independent predictor of cardiovascular mortality [16]. In the Dallas Heart Study, SDMA was found to be associated with an increase in aortic wall thickness [16], and in the SABPA study, with an increase in carotid intima-media thickness [18].

Given that dronedarone produces a regression of the LVH induced by hypertension, we hypothesize that this drug could improve thoracic aortic remodeling. The aim of the present study is to test this hypothesis, assessing the following in SHRs: 1) the effect of dronedarone on the structure of the thoracic aortic wall, 2) the effect of dronedarone on the passive mechanical function of the aorta, and 3) the role of SDMA in the regression of aortic remodeling mediated by dronedarone.

## Materials and methods

Experiments were performed using spontaneously hypertensive rats (SHR) from the colony maintained at the Animal House facility of the Universidad Autónoma de Madrid. All experimental procedures conformed to the Guidelines for the Care and Use of Laboratory Animals and the Spanish legislation (Directive 2010/63/UE and RD 53/2013) and were approved by the Ethics Review Board of Hospital Universitario Gregorio Marañón and of the local Government (Comunidad Autónoma de Madrid).

### Animals and experimental protocols

The rats were supplied with standard rat chow and drinking water *ad libitum*. They were maintained on a 12h/12h light/dark cycle and housed at a constant temperature of 24°C and relative humidity of 40%.

Ten-month-old male SHRs were randomly assigned to an intervention group (SHR-D, n = 8), where they received oral dronedarone (Multaq, Sanofi-Aventis, Barcelona, Spain) (100 mg/kg, once daily) for a period of 14 days, or to a control group (SHR, n = 8), where they were given the vehicle only, or to a group (SHR-A, n = 8) where they were given amiodarone (Trangorex, Sanofi-Aventis, Barcelona, Spain) (30 mg/kg, once daily). A fourth group of normotensive control rats (Wistar-Kyoto rats, WKY, n = 8) was also added. Once treatment was complete, rats were sedated with an intraperitoneal injection of diazepam (Valium, Roche Pharmaceuticals, Madrid, Spain) (4 mg/kg) and ketamine (Ketolar, Parke-Davis, Madrid, Spain) (10 mg/kg) and killed by decapitation. The thoracic aorta was excised to study its vascular structure, mechanical function and the biomarker SDMA.

### Arterial pressure and heart rate measurements

Systolic arterial pressure (SAP) and heart rate (HR) were measured in conscious WKY, SHR, SHR-D and SHR-A animals with a photoelectric sensor (Niprem 546, Cibertec, Madrid, Spain) using the tail-cuff method. Several determinations were made, and the findings were considered valid if 10 consecutive measurements were within 10 mmHg of each other.

### Vascular structure: Histology and confocal microscopy

#### Histological study

*T*he study of aortic geometry was performed as previously described [19]. A 1 mm segment of the thoracic aorta was fixed in 4% sodium-buffered formaldehyde. Samples were then dehydrated and embedded in paraffin. Serial sections (5 μm) were stained with orcein. A total of 8 segments of thoracic aorta per group were observed and analyzed using a high-resolution camera (Sony CCD IRIS) attached to a microscope (Leica DMLB, 4x objective). The external diameter (ED) (inner diameter + tunica intima + tunica media + tunica adventitia) and lumen diameter (LD) of the aorta were measured. The wall thickness (WT) was expressed as (ED–LD)/2, the wall-to-lumen ratio (W/L) as (WT/LD) × 100, and the cross-sectional area (CSA) (tunica intima + tunica media + tunica adventitia) as (π/4) × (ED2−LD2) [20]. The volume density of the elastic fibers in the tunica media was analyzed in 8 segments of thoracic aorta per group (40x objective). The morphometric analysis was performed using the method of Gundersen et al. [21].

#### Confocal microscopy study

Confocal microscopy was used to assess the thickness of the tunica media and tunica adventitia in the thoracic aorta as previously described [19]. Briefly, segments (1 mm in length) were fixed in 4% PFA before being washed in 9% saline solution and stained with DAPI (1:500 from a stock solution of 5 mg/mL). One ring and one longitudinal section were cut from each segment and mounted on a slide with a small well made of spacers to avoid vessel compression. The well was then filled with the mounting medium (Citifluor, Aname, Spain). The rings were visualized with a 20x objective at zoom 2 at the 488 nm/515 nm line, and several images were captured to quantify the thickness of the media in the ring section. The longitudinal section was mounted with the tunica adventitia facing upwards and viewed using a Leica TCS SP2 confocal microscope (Leica Microsystems, Wetzlar, Germany) at excitation 405 nm/emission 410–475 nm. In each artery, three randomly selected regions were visualized with a 20x objective at zoom 4. In each of these regions, stacks of 1 μm thick serial optical sections were captured from the tunica adventitia. Quantitative analysis was performed using MetaMorph Image Analysis Software (Universal Imaging, Co., UK) as previously described [19].

### The passive mechanical function of the aorta

The study of the passive mechanical function of the aorta was performed as previously described [19]. Briefly, 3 mm-long descending thoracic aortic rings were suspended on two intraluminal parallel wires, placed into an organ bath containing oxygenated calcium-free (0Ca^2+^) Krebs-Henseleit solution (KHS, in mM: 115.0 NaCl, 25.0 NaHCO_3_, 4.7 KCl, 1.2 MgSO_4_, 7 H_2_O, 1.2 KH_2_PO_4_, 11.1 glucose and 10 EGTA), and connected to a Piodem strain gauge for isometric tension recording. The vessel mounted on the wire support was then left for 30 min to equilibrate at 37°C before undergoing 200 μm stretches using a micrometer to measure the tension applied.

Internal circumferential (L) and circumferential wall tension (T) were calculated using the following equations, reported elsewhere [22]:

L=(π+2)d+2f (1)

T=F/2g (2)

where d (mm) is the diameter of the wires, f (mm) is the separation between the wires (increasing by micrometer intervals), F (N) is the wall force, and g (mm) is the length of the vessel.

The experimental values F_i_ and f_i_ (for each stretch) were used to calculate L_i_ and T_i_ according to Eqs (1) and (2) above, and the values of T_i_ and L_i_ were fitted by an exponential equation using non-linear regression analysis:

$$T_{i}=Ae^{BL_{i}}$$ (3)

The wall stiffness parameter (B) was used to compare the passive mechanical properties of the aorta in the WKY, SHR,SHR-D and SHR-A groups.

### Measurement of symmetric dimethylarginine concentrations in the aorta

Mass spectrometric determination of SDMA concentrations was performed as previously described using a fully validated high-throughput liquid chromatography/tandem mass spectrometry (LC-MS/MS) assay [23,24]. In brief, samples were analyzed using 96-well 0.20-μm microfiltration plates pre-coated with internal standards. After conversion to their butyl ester derivatives, analytes were evaluated using a Varian 1200L Triple Quadrupole MS (Varian, Walnut Creek, CA, USA) in the positive electrospray ionization (ESI+) mode.

### Statistical analyses

Statistical analyses were performed using GraphPad Prism (Version 5) and SPSS (Version 20). The data are expressed as mean ± SEM. Statistical differences between the groups (physiological, structural and mechanical parameters, and concentrations of SDMA) were analyzed by one-way ANOVA. A post hoc Bonferroni correction was applied. Non-regression analysis with an exponential equation was used to estimate mechanical parameters (the B parameter). The Pearson coefficient was applied to analyze the correlation between aortic SDMA concentrations and wall thickness. P<0.05 was considered to be statistically significant.

## Results

### Dronedarone and physiological parameters

Rat weight, systolic arterial pressure and heart rate are shown in Table 1. Rat weight was higher in the WKY group (11.4%, P<0.01) than in the SHR group, although no differences were detected between the SHR, SHR-D and SHR-A groups. The administration of dronedarone to the SHR-D group for two weeks was associated with a reduction in tail-cuff SAP (18.6%, P<0.01) when compared to the SHR group, but no differences were detected with respect to the WKY group. The decrease in SAP found in the SHR-D group was associated with a reduction in HR when compared to both SHR and WKY groups (20.3%, P<0.001 and 21.3%, P<0.001, respectively). The SHR-A group showed the same level of SAP reduction and HR as the SHR-D group.

**Table 1:** Weight, arterial pressure and heart rate of WKY, SHR, SHR-D and SHR-A groups.

	WKY (n = 8)	SHR (n = 8)	SHR-D (n = 8)	SHR-A (n = 8)
**Body weight (g)**	441.13 ± 10.21	390.92 ± 5.12**	387.85 ± 3.82**	385.14 ± 3.15**
**SAP (mmHg)**	138 ± 15	178 ± 20**	145 ± 10^##^	143 ± 20^##^
**HR (bpm)**	400 ± 24	395 ± 17	315 ± 14***^,^^###^	324 ± 21***^,^^###^

SAP: systolic arterial pressure; HR: heart rate; WKY: Wistar-Kyoto rats; SHR: spontaneously hypertensive rats; SHR-D: spontaneously hypertensive rats treated with dronedarone; SHR-A: spontaneously hypertensive rats treated with amiodarone. Statistically significant differences between WKY, SHR and SHR-D are shown as follows:

**P<0.01 vs. WKY,

***P<0.001 vs. WKY,

^##^ P<0.01 vs. SHR,

^###^P<0.001 vs. SHR. Values are given as mean ± SEM.

### Dronedarone improves structural aortic remodeling

**Fig 1:**
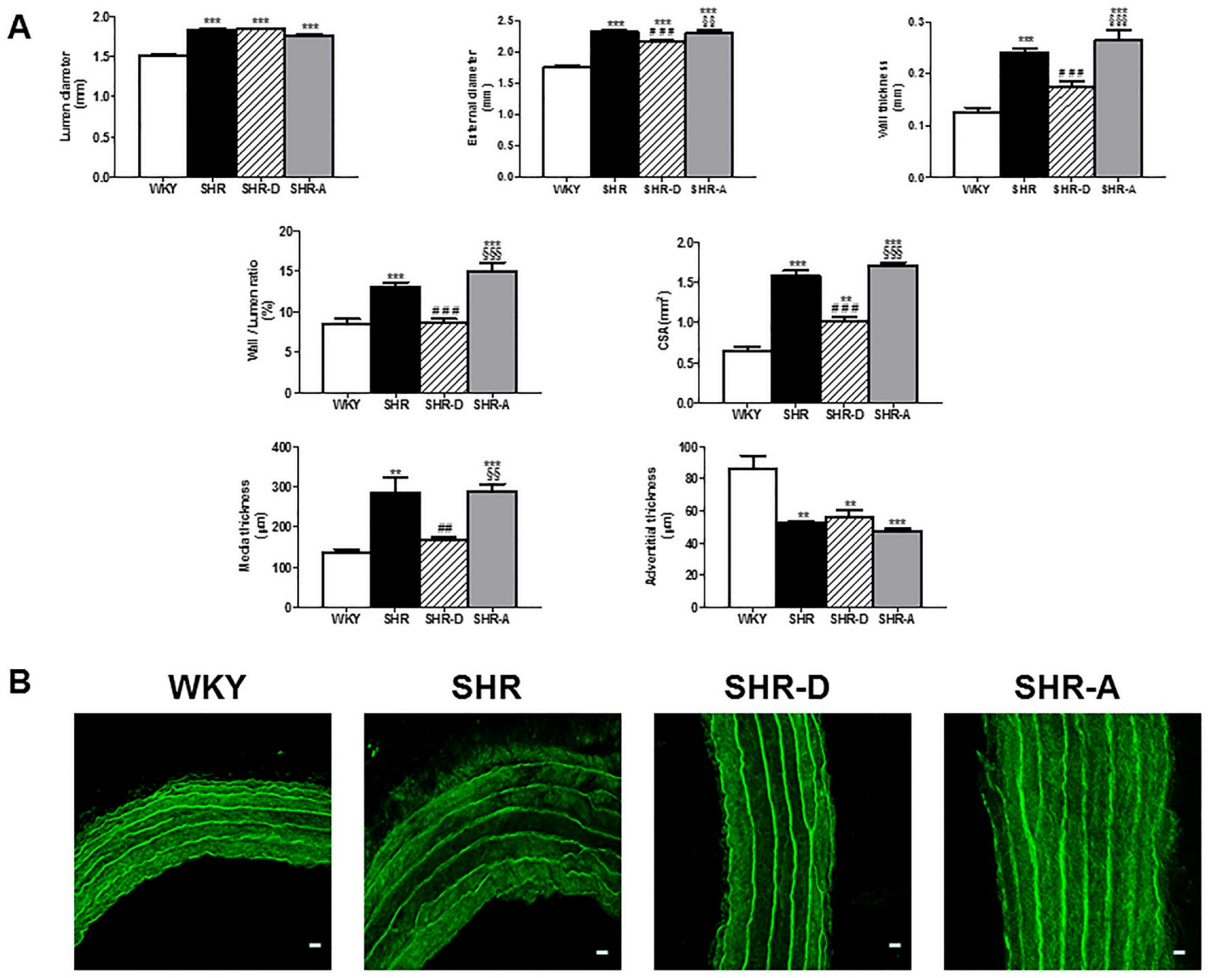
Aortic geometry obtained by histology and confocal microscopy. (A) Aortic structural parameters: lumen diameter, external diameter, wall thickness, wall/lumen ratio and cross-sectional area (obtained by histology); tunica media and adventitia thickness (obtained by confocal microscopy) in WKY (Wistar-Kyoto rats), SHR (spontaneously hypertensive rats), SHR-D (spontaneously hypertensive rats treated with dronedarone) and SHR-A (spontaneously hypertensive rats treated with amiodarone). Statistically significant differences between the WKY, SHR, and SHR-D and SHR-A groups are shown as follows: **P<0.01 vs. WKY, ***P<0.001 vs. WKY, ^##^P<0.01 vs. SHR, ^###^ P<0.001 vs. SHR, ^§§^P<0.01 vs SHR-D, ^§§§^P<0.001 vs SHR-D. Values are given as mean ± SEM. n = 8 rats per group. (B) Examples of the tunica media obtained using confocal microscope images (20 x at zoom 2, 100 μm) from WKY, SHR, SHR-D and SHR-A animals.

**Fig 2:**
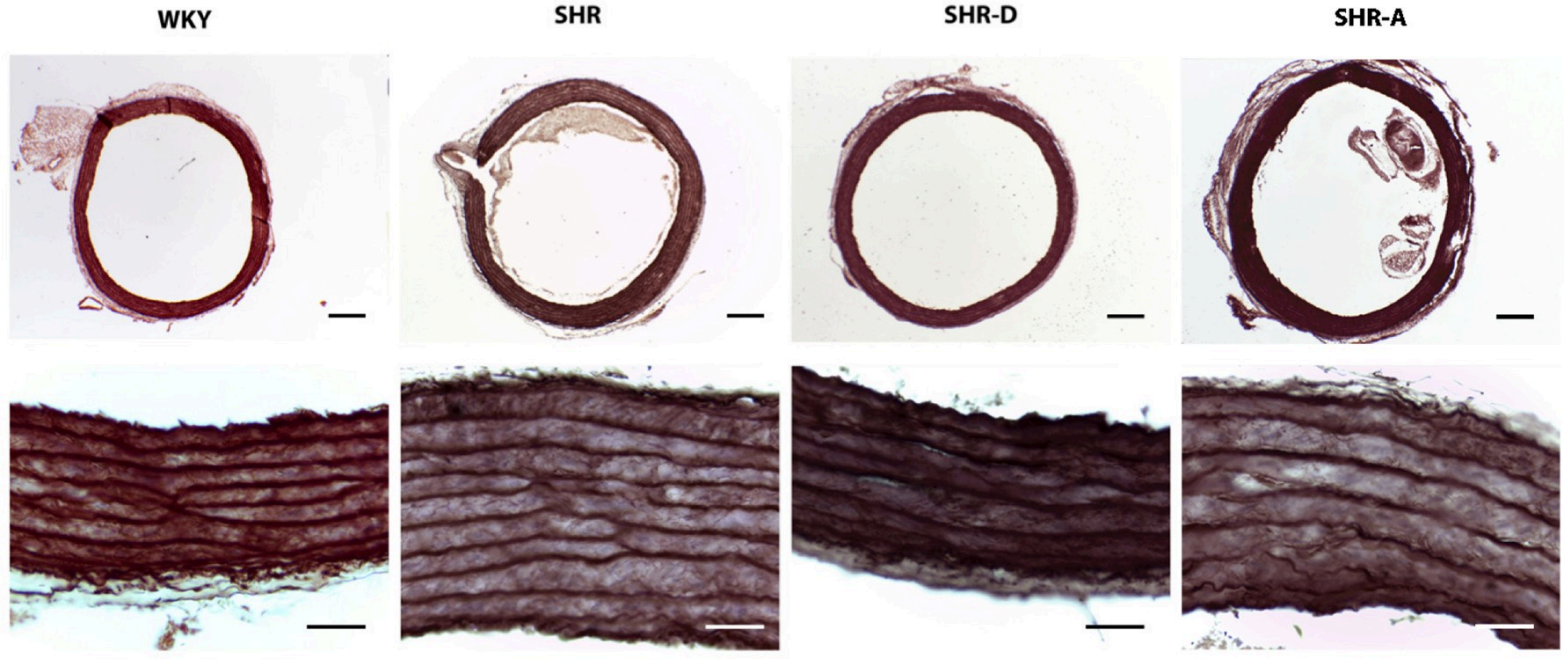
Sections of aorta and elastic fibers obtained from the histological study. (A) Examples of sections of aorta (tunica intima + media + adventitia) (Orcein 40 x, 300 μm) and (B) examples of the elastic fiber content of the tunica media (Orcein 400 x, 50 μm) from WKY (Wistar-Kyoto rats), SHR (spontaneously hypertensive rats), SHR-D (spontaneously hypertensive rats treated with dronedarone) and SHR-A (spontaneously hypertensive rats treated with amiodarone).

The aortic geometric parameters obtained from the histological study and the confocal microscopy are shown in Figs 1A, 1B and 2A (S1 Table). The SHR group showed outward hypertrophic remodeling associated with an increase in LD (21.8%, P<0.001) and CSA (arterial wall mass) (142.2%, P<0.001) in comparison to the WKY group. The administration of dronedarone to the SHR-D group resulted in a 35.7% decrease in CSA (P<0.001) when compared to the SHR group. No differences in LD were detected between the SHR and SHR-D groups. The WT and ED of the SHR group increased by 94.4% (P<0.001) and 31.7% (P<0.001), respectively, when compared to the WKY group; however, two weeks of dronedarone administration decreased these parameters by 27% (P<0.001) and 6.7% (P<0.001) in the SHR-D group, and no differences were detected in WT with respect to the WKY group. The SHR group presented an increase in W/L (55.5%, P<0.001) when compared to the WKY group. In the SHR-D group, dronedarone produced a decrease in W/L (34%, P<0.001) with respect to the SHR group, and no differences were detected with respect to the WKY group. All structural parameters were similar in the SHR and SHR-A groups.

When compared to the WKY group, the media thickness of the SHR group was increased by 128.6% (P<0.01). The analysis by confocal microscopy shows that dronedarone produced a marked decrease in the thickness of this tunica (41.4%, P<0.01) when comparing the SHR-D group to the SHR group, and that this value was comparable to that of the WKY group. No significant differences in adventitial thickness were observed between the SHR and SHR-D groups. Both SHR and SHR-D animals showed a decrease in this parameter (38.2%, P<0.01; 34.5%, P<0.01, respectively) when compared to the WKY group. The media and adventitial thickness were similar in the SHR and SHR-A groups (S1 Table).

### Dronedarone improves the mechanical properties of the aorta

**Fig 3:**
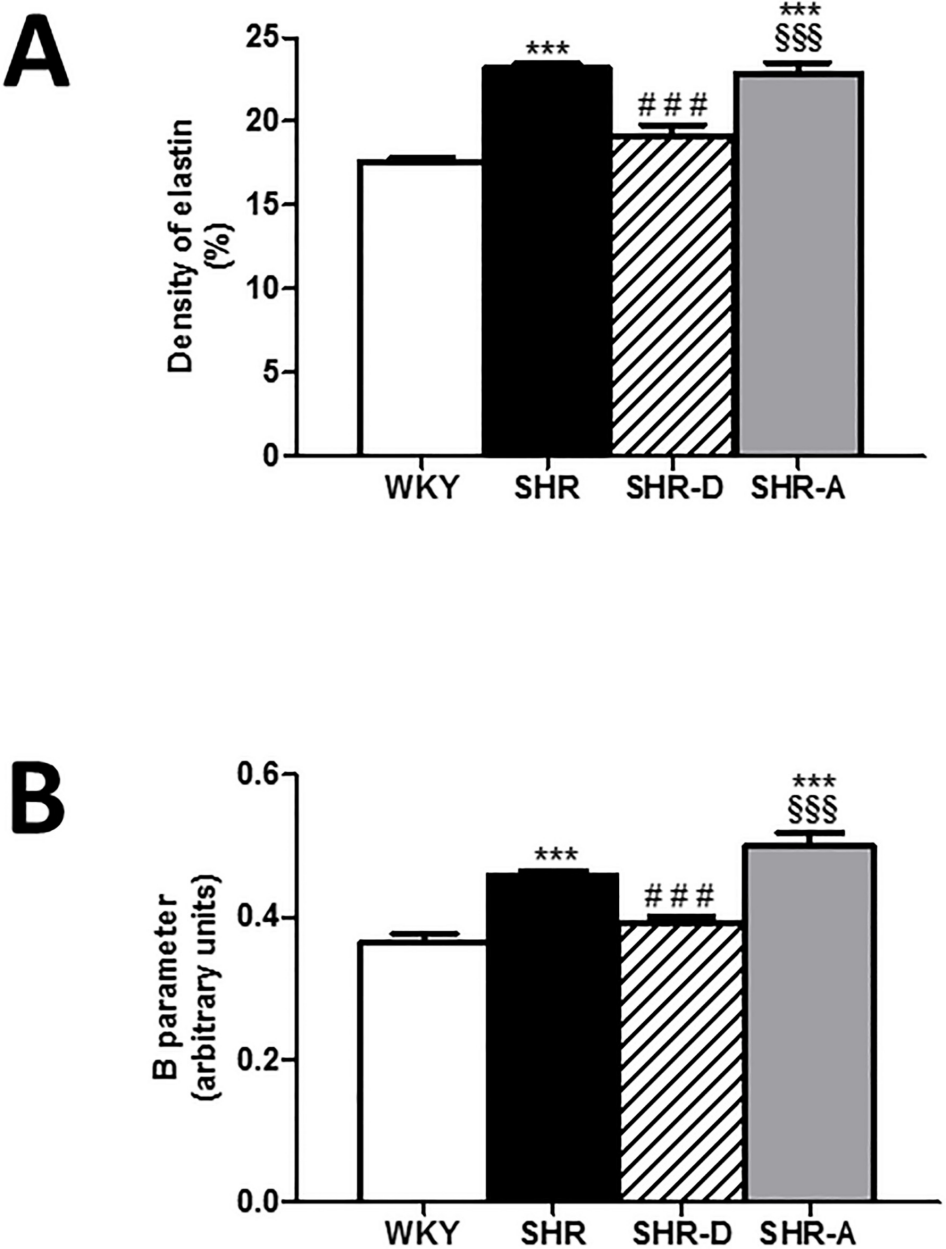
Aortic elastin density and wall stiffness in all experimental groups. (A) Elastin density and (B) the passive mechanical properties (B parameter) of the aorta in WKY (Wistar-Kyoto rats), SHR (spontaneously hypertensive rats), SHR-D (spontaneously hypertensive rats treated with dronedarone) and SHR-A (spontaneously hypertensive rats treated with amiodarone). Statistically significant differences between WKY, SHR, SHR-D and SHR-A are shown as follows: ***P<0.001 vs. WKY, ^###^ P<0.001 vs. SHR, ^§§§^P<0.001 vs SHR-D. Values are given as mean ± SEM. n = 8 rats per group.

The volume density of the elastic fibers in the tunica media obtained from the histological study is shown in Figs 2B and 3A (S1 Table). In the SHR group, the volume density of these fibers was increased by 32.1% (P<0.001) compared to the WKY group. Dronedarone administration resulted in a 17.4% decrease in this parameter in the SHR-D group (P<0.001). No differences were found between the WKY and SHR-D groups. The volume density of the elastic fibers was similar in the SHR and SHR-A groups.

The tensile forces acting on the aortic wall are shown in Fig 3B (S1 Table). Experimental data for the circumferential wall tension–internal circumference curve were fitted to an exponential model to calculate the B parameter, which is related to the slope of the above curve. In the SHR group, the B parameter was increased (50.5%, P<0.001) compared to the WKY group. The B parameter in the SHR-D group was smaller (13.9%, P<0.001) than that of the SHR group. No differences were found between the WKY and SHR-D groups. The B parameter was similar in the SHR and SHR-A groups.

### Dronedarone decreases aortic symmetric dimethylarginine concentration

**Fig 4:**
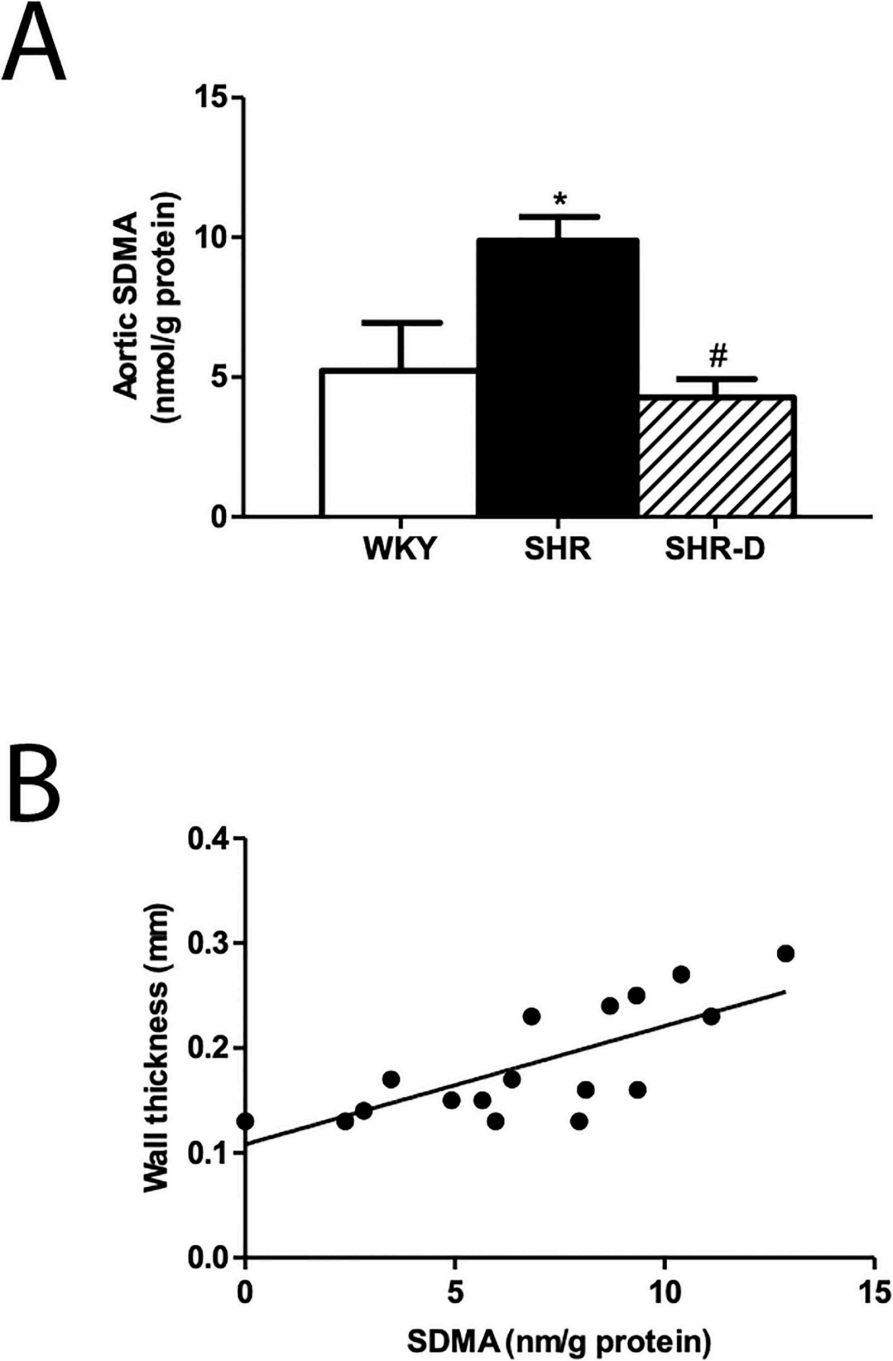
Aortic symmetric dimethylarginine concentration and its association with wall thickness. (A) Aortic SDMA concentration in WKY (Wistar-Kyoto rats), SHR (spontaneously hypertensive rats) and SHR-D (spontaneously hypertensive rats treated with dronedarone). Statistically significant differences between the WKY, SHR and SHR-D groups are shown as follows: *P<0.05 vs. WKY, ^#^ P<0.05 vs. SHR. Values are given as mean ± SEM. n = 8 rats per group. (B) Pearson’s correlation analysis shows a positive correlation (P<0.001) between aortic SDMA concentration and wall thickness in all experimental groups.

The aortic SDMA concentration is shown in Fig 4A (S1 Table). SDMA in the SHR group was increased by 88.7% (P<0.05) compared to the WKY group. The administration of dronedarone to the SHR-D group decreased SDMA by 56.8% (P<0.05) when compared to the SHR group; in addition, the aortic SDMA value returned to normal after treatment with dronedarone.

A correlation analysis showed a positive correlation between aortic SDMA concentration and wall thickness (Pearson’s r = 0.76, P<0.001) in all experimental groups (WKY, SHR and SHR-D) (Fig 4B).

## Discussion

The present study was performed to evaluate the effect of dronedarone on the regression of aortic remodeling after two weeks of treatment. Our main findings are that: 1) dronedarone produces changes in the aortic structure (decreased wall thickness); 2) dronedarone produces changes in the mechanical properties of the aorta (reduced volume density of the elastic fibers and decreased wall stiffness); 3) these effects could be associated with a reduction in aortic SDMA concentrations; and 4) Amiodarone (antiarrhythmic agent) does not produce changes in the structure of the aorta or in the mechanical properties.

### Dronedarone and the regression of aortic remodeling

Dronedarone is a new antiarrhythmic developed for the treatment of atrial fibrillation and atrial flutter [25]. It exerts its antiarrhythmic effects through multichannel blockade of sodium, potassium, and calcium channels and exhibits antiadrenergic properties [26]. Dronedarone reduces the risk of acute coronary syndrome because it reduces the heart rate and arterial pressure, and it has a direct cardioprotective effect (resulting in a reduction in infarct size in an animal ischemia/reperfusion model) [11]. This cardioprotective effect has been associated with reduced mortality [27]. Our results are consistent with other studies that describe a decrease in blood pressure and heart rate associated with the administration of this drug [11]. Our group has previously demonstrated that dronedarone produces a regression of LVH in hypertensive rats after two weeks of treatment [14]. However, the impact of this drug on the remodeling of the elastic arteries (e.g. the aorta) has not yet been studied.

Cardiovascular disease is the leading cause of death in the general population [28]. Hypertension induces morphological and physiological changes in the arterial wall [29]; therefore, the regression of these changes is one of the goals of antihypertensive therapy [7] in order to reduce the incidence of adverse cardiovascular events [5,6]. Several studies have shown changes in aortic remodeling (a reduction in aortic wall thickness) in SHRs as a result of antiarrhythmic and antihypertensive therapy (β-adrenergic blockers, ACE inhibitors, angiotensin receptor antagonists, and calcium channel blockers) [19,30–34]. When compared to these studies, dronedarone did not show any differential effects on aortic remodeling (decreased aortic media layer and CSA); however, dronedarone also produced changes in the passive mechanical properties of the aorta. Increases in wall stress and biomechanical stretch are a consequence of hypertension and are potent drivers of arterial remodeling [35]. These alterations are also linked to a higher risk of adverse cardiovascular events. We therefore found it to be of interest to study the effects of dronedarone treatment on the structure and mechanical properties of the elastic arteries (the aorta and carotid artery). We and other authors have previously observed that SHRs show outward aortic hypertrophic remodeling, increased elastic fiber content and increased wall stiffness [19,36]. This is a consequence of arterial hypertension in the elastic arteries [3,37]. Under our experimental conditions, dronedarone reduced the volume density of the elastic fibers, and decreased wall stiffness after two weeks of treatment. Similar results have been described with antihypertensive therapy (angiotensin receptor antagonists), but after four months of treatment.

### The association between dronedarone, aortic wall thickness and symmetric dimethylarginine concentration

Our results show that dronedarone reduces the aortic concentration of SDMA. SDMA is an inhibitor of the intracellular uptake of L-arginine, and may additionally affect vascular homeostasis by NO-independent mechanisms [15,28]. SDMA is found in the plasma and in all human and rodent tissues [17]. Several studies have identified SDMA as potential biomarker for cardiovascular disease, associated with major cardiovascular events and mortality in patients with coronary artery disease, peripheral arterial disease and end-stage renal disease [38–41]. In addition, SDMA is predictive of all-cause mortality after cerebral ischemic stroke [42,43].

High plasma SDMA concentrations have been positively associated with vascular wall thickness [16,18,44,45]. In the Dallas Heart Study, SDMA was associated with increased aortic wall thickness in a general population-based cohort [16]. One experimental study has shown that SDMA may contribute to proinflammatory events in the vascular wall by opening store-operated calcium channels in monocytes, leading to monocyte activation. This mechanism could be involved in the association between SDMA concentration and vascular wall thickness [28].

Our experimental study found a positive association between SDMA concentration and thoracic aortic wall thickness, which may support these clinical observations. Thus, our results not only support the regression of aortic remodeling after treatment with dronedarone, but also suggest that a decrease in SDMA levels may be related to this effect. We cannot explain the molecular mechanisms responsible for the effect of dronedarone on aortic remodeling; however, our results suggest an important role for the lowering of SDMA concentration in strategies to delay vascular remodeling.

### Dronedarone and stroke

The deleterious effects of hypertension include arterial remodeling (increased wall thickness) [29], and a single-vessel occlusion can significantly reduce the blood flow in ischemia [46]. Hypertension is the leading cause of stroke. In the ATHENA study (a randomized double-blind clinical trial), dronedarone reduced the risk of stroke by 36% in 2301 patients receiving dronedarone (1999 with hypertension) when compared to 2327 patients on a placebo (1996 with hypertension) [10]. The mechanisms by which dronedarone might reduce the risk of stroke are a reduction in blood pressure and a decrease in heart rate [10]. Several studies have shown that SDMA is associated with increased carotid wall thickness [18,44,45] and an increase in cardiovascular events (stroke) [42,43]. Therefore, as the present study shows that dronedarone reduces vascular wall thickness and decreases SDMA concentration, it is reasonable to speculate that its effect on vascular remodeling might be the mechanism by which dronedarone reduces the risk of stroke. Further studies are necessary to confirm this hypothesis.

## Conclusions and perspectives

Regarding our initial hypothesis, we conclude that dronedarone improves the structure and the passive mechanical properties of the thoracic aorta in hypertensive rats, and that this effect could be associated with the reduction in aortic SDMA concentrations. Dronedarone is currently indicated for the treatment of atrial fibrillation; however, if the results of this study are confirmed in humans, dronedarone could be taken into consideration for the treatment of patients with atrial fibrillation and chronic hypertension.

## Supporting information

S1 Table Geometry, mechanical properties and symmetric dimethylarginine of the aorta in WKY, SHR, SHR-D and SHR-A. (PDF)
