# Supplementary material for: The protective effect of dronedarone on the structure and mechanical properties of the aorta in hypertensive rats by decreasing the concentration of symmetric dimethylarginine (SDMA)
Source: PLoS One. 2019 May 21;14(5):e0216820. doi: 10.1371/journal.pone.0216820 (PMC6529158; doi:10.1371/journal.pone.0216820)
Supplement: S1 Table — (PDF) [file pone.0216820.s001.pdf]

S1 Table. Geometry, mechanical properties and symmetric dimethylarginine of the aorta in WKY, SHR, SHR-D and SHR-A

	WKY	SHR	SHR-D	SHR-A
LD (mm)	1.50 ± 0.01	1.83 ± 0.01***	1.84 ± 0.01***	0.26 ± 0.01***
ED (mm)	1.76 ± 0.01	2.32 ± 0.02***	2.16 ± 0.01***,###	2.29 ± 0.05***,§§
WT (mm)	0.12 ± 0.009	0.24 ± 0.009***	0.16 ± 0.007###	0.26 ± 0.01***,§§§
W/L (%)	8.44 ± 0.64	13.13 ± 0.51***	8,68 ± 0.40###	15.02 ± 1.04***,§§§
CSA (mm^2)	0.65 ± 0.04	1.57 ± 0.07***	1.01 ± 0.04**,###	1.70 ± 0.14***,§§§
Media thickness (µm)	134.33 ± 9.89	284.27 ± 37.83**	166.86 ± 7.91##	287.96 ± 8.33***,§§
Adventitia thickness (µm)	86.18 ± 8.29	52.33 ± 1.49**	56.46 ± 4.21**	47 ± 1.81***
Volume density elastic fibers (%)	17.52 ± 0.28	23.15 ± 0.30***	16.72 ± 0.42###	22.87 ± 0.76***,§§§
B Parameter	0.36 ± 0.01	0.45 ± 0.006***	0.39 ± 0.009###	0.5 ± 0.01***,§§§
SDMA (nm/g protein)	5.23 ± 1.71	9.87 ± 0.85*	4.25 ± 0.65#	----------

LD: lumen diameter; ED: external diameter; WT: Wall thickness; W/L: wall/lumen ratio; CSA: cross-sectional area; SDMA: symmetric dimethylarginine, in WKY Wistar-Kyoto rats), SHR (spontaneously hypertensive rats), SHR-D (spontaneously hypertensive rats treated with dronedarone) and SHR-A (spontaneously hypertensive rats treated with amiodarone). Statistically significant differences between the WKY, SHR, SHR-D and SHR-A groups are shown as follows: *P<0.05 vs. WKY, **P<0.01 vs. WKY, ***P<0.001 vs. WKY, # P<0.05 vs. SHR, ##P<0.01 vs. SHR, ### P<0.001 vs. SHR, §§P<0.01 vs SHR-D, §§§P<0.001 vs SHR-D. Values are given as mean ± SEM. n=8 rats per group. Statistical differences between the groups were analyzed by one-way ANOVA.
